# Bevacizumab, Irinotecan, or Topotecan Added to Temozolomide for Children With Relapsed and Refractory Neuroblastoma: Results of the ITCC-SIOPEN BEACON-Neuroblastoma Trial

**DOI:** 10.1200/JCO.23.00458

**Published:** 2024-01-08

**Authors:** Lucas Moreno, Rebekah Weston, Cormac Owens, Dominique Valteau-Couanet, Marion Gambart, Victoria Castel, C. Michel Zwaan, Karsten Nysom, Nicolas Gerber, Aurora Castellano, Genevieve Laureys, Ruth Ladenstein, Jochen Rössler, Guy Makin, Dermot Murphy, Bruce Morland, Sucheta Vaidya, Estelle Thebaud, Natasha van Eijkelenburg, Deborah A. Tweddle, Giuseppe Barone, Julie Tandonnet, Nadege Corradini, Pascal Chastagner, Catherine Paillard, Francisco J. Bautista, Soledad Gallego Melcon, Bram De Wilde, Lynley Marshall, Juliet Gray, Susan A. Burchill, Gudrun Schleiermacher, Louis Chesler, Andrew Peet, Martin O. Leach, Kieran McHugh, Roisin Hayes, Neil Jerome, Hubert Caron, Jennifer Laidler, Nicola Fenwick, Grace Holt, Veronica Moroz, Pamela Kearns, Simon Gates, Andrew D.J. Pearson, Keith Wheatley

**Affiliations:** ^1^ Vall d’Hebron University Hospital, Barcelona, Spain; ^2^ University of Birmingham, Birmingham, United Kingdom; ^3^ Children's Hospital Ireland, Dublin, Ireland; ^4^ Institut Gustave Roussy, Paris, France; ^5^ Hôpital des Enfants, Bordeaux, France; ^6^ Hospital Universitario La Fe, Valencia, Spain; ^7^ Princess Maxima Center, Utrecht, the Netherlands; ^8^ Rigshospitalet, Copenhagen, Denmark; ^9^ Universitats-Kinderspital, Zurich, Switzerland; ^10^ Bambino Gesù Children's Hospital, Rome, Italy; ^11^ University Hospital Gent, Gent, Belgium; ^12^ St Anna Kinderspital, Vienna, Austria; ^13^ Inselspital, Universitätsspital Bern, Bern, Switzerland; ^14^ Central Manchester and Manchester Children's University Hospitals NHS Trust, Manchester, United Kingdom; ^15^ NHS Greater Glasgow and Clyde, Glasgow, United Kingdom; ^16^ Birmingham Women's and Children's NHS Foundation Trust, Birmingham, United Kingdom; ^17^ The Royal Marsden NHS Foundation Trust & Institute for Cancer Research, London, United Kingdom; ^18^ Hôpital Mère-Enfant, Nantes, France; ^19^ The Newcastle upon Tyne Hospitals NHS Foundation Trust, Newcastle, United Kingdom; ^20^ Great Ormond Street Hospital, London, United Kingdom; ^21^ Hôpital des Enfants, Hémato-Oncologie, Bordeaux, France; ^22^ Centre Léon Bérard, Lyon, France; ^23^ Hôpital d'Enfants, Nancy, France; ^24^ Hôpital de Hautepierre, Strasbourg, France; ^25^ University Hospital Southampton, Southampton, United Kingdom; ^26^ St James University Hospital, Leeds, United Kingdom; ^27^ Institute Curie, Paris, France; ^28^ Roche, Basel, Switzerland

## Abstract

**PURPOSE:**

Outcomes for children with relapsed and refractory high-risk neuroblastoma (RR-HRNB) remain dismal. The BEACON Neuroblastoma trial (EudraCT 2012-000072-42) evaluated three backbone chemotherapy regimens and the addition of the antiangiogenic agent bevacizumab (B).

**MATERIALS AND METHODS:**

Patients age 1-21 years with RR-HRNB with adequate organ function and performance status were randomly assigned in a 3 × 2 factorial design to temozolomide (T), irinotecan-temozolomide (IT), or topotecan-temozolomide (TTo) with or without B. The primary end point was best overall response (complete or partial) rate (ORR) during the first six courses, by RECIST or International Neuroblastoma Response Criteria for patients with measurable or evaluable disease, respectively. Safety, progression-free survival (PFS), and overall survival (OS) time were secondary end points.

**RESULTS:**

One hundred sixty patients with RR-HRNB were included. For B random assignment (n = 160), the ORR was 26% (95% CI, 17 to 37) with B and 18% (95% CI, 10 to 28) without B (risk ratio [RR], 1.52 [95% CI, 0.83 to 2.77]; *P* = .17). Adjusted hazard ratio for PFS and OS were 0.89 (95% CI, 0.63 to 1.27) and 1.01 (95% CI, 0.70 to 1.45), respectively. For irinotecan ([I]; n = 121) and topotecan (n = 60) random assignments, RRs for ORR were 0.94 and 1.22, respectively. A potential interaction between I and B was identified. For patients in the bevacizumab-irinotecan-temozolomide (BIT) arm, the ORR was 23% (95% CI, 10 to 42), and the 1-year PFS estimate was 0.67 (95% CI, 0.47 to 0.80).

**CONCLUSION:**

The addition of B met protocol-defined success criteria for ORR and appeared to improve PFS. Within this phase II trial, BIT showed signals of antitumor activity with acceptable tolerability. Future trials will confirm these results in the chemoimmunotherapy era.

## INTRODUCTION

Neuroblastoma remains one of the main causes of death from childhood cancer. Despite many targeted and novel therapies being evaluated over the past decades and improvements in frontline therapy, long-term outcomes remain poor, with 5-year survival below 20% for children with refractory/relapsed disease.^1-5^

**CONTEXT**

**Key Objective**
New therapeutic combinations are urgently needed for children with relapsed and refractory neuroblastoma.
**Knowledge Generated**
The trial used a novel factorial multiarm multistage platform design to test multiple questions and has become the largest randomized trial in this setting. Three temozolomide (T)-based chemotherapy regimens and the addition of the anti–vascular endothelial growth factor bevacizumab (B) were tested.
**Relevance *(S. Bhatia)***
The addition of B to T-based chemotherapy improved best overall response rate in patients with high-risk relapsed/refractory neuroblastoma. An interaction between irinotecan and B is providing evidence for future studies that will compare irinotecan-T and B with a chemo-immunotherapy regimen in this population.*
  *Relevance section written by *JCO* Associate Editor Smita Bhatia, MD, MPH, FASCO.

Second-line chemotherapy regimens have been evaluated over the past 20 years reporting a wide range of response rates (0%-64%^2,6,7^), with little data about survival outcomes and, importantly, lack of clarity regarding comparative benefits, with only one trial being randomized.^8^ Cooperative groups have developed several temozolomide (T)-based backbone regimens where novel drugs could be added.^9-11^

Antiangiogenic therapies are used in multiple adult cancer indications, although, to our knowledge, to date, none has demonstrated clear benefit in pediatric cancers.^12-14^ Preclinical in vitro and in vivo data of bevacizumab (B; a monoclonal antibody against vascular endothelial growth factor [VEGF]) as single agent and in combination, including irinotecan-temozolomide (IT) as well as other anti-VEGF therapies, support the evaluation of antiangiogenic agents in neuroblastoma.^15-22^ B is among the most established of antiangiogenic drugs and has gained approval in many adult indications.

The BEACON-Neuroblastoma trial opened in 2013 with two main aims: to identify the best backbone chemotherapy regimen on which to add new targeted therapies (T, IT, or topotecan-temozolomide [TTo]) and to establish the role of the addition of B in children with refractory/relapsed neuroblastoma within a randomized phase II trial.

## MATERIALS AND METHODS

### Patients

Eligible patients were age 1 to 21 years with confirmed diagnosis of high-risk neuroblastoma. Disease status was relapsed or refractory to frontline therapy. Refractory disease was defined as lack of adequate response to frontline therapy using European Neuroblastoma Research Network (SIOPEN) criteria used at the time of enrollment: persistence of three or more spots on metaiodobenzylguanidine (I^123^ MIBG) scan (with or without persistence of bone marrow disease). Measurable (as per RECIST 1.1^23^) or evaluable disease (uptake on I^123^ MIBG scan) was required. Previous treatment with T, I, or B was not allowed. Patients with only bone marrow detectable disease were not eligible.

Eligible patients had adequate performance status, organ and bone marrow function, and appropriate wash out periods for previous therapies. Full eligibility criteria are provided in the trial protocol, available as Data Supplement (online only). All patients, parents, and/or legal guardians provided written informed consent. Assent was sought from minors where appropriate.

### Trial Design and Interventions

**FIG 1.**
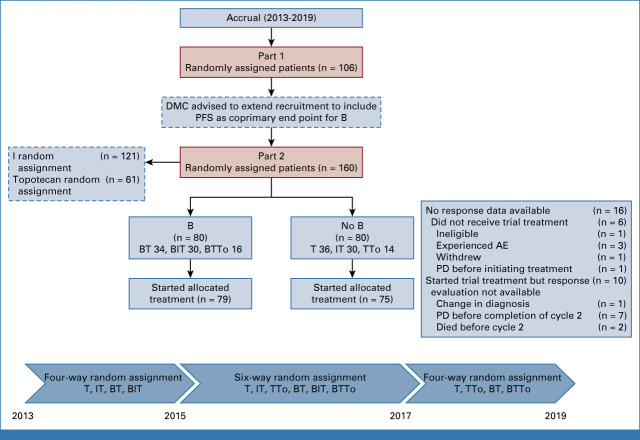
CONSORT diagram of the BEACON Neuroblastoma trial. AE, adverse event; B, bevacizumab; BT, bevacizumab-temozolomide; BIT, bevacizumab-irinotecan-temozolomide; BTTo, bevacizumab-topotecan-temozolomide; DMC, Data Monitoring Committee; I, irinotecan; IT, irinotecan-temozolomide; PD, progressive disease; PFS, progression-free survival; T, temozolomide; TTo, topotecan-temozolomide.

BEACON-Neuroblastoma (ITCC-032; ClinicalTrials.gov identifier: NCT02308527; EudraCT 2012-000072-42) was an open-label, randomized phase II trial. The trial was started with a 2 × 2 factorial design, in which patients were randomly assigned to two chemotherapy regimens (T alone or IT), with or without B (bevacizumab-temozolomide [BT] and bevacizumab-irinotecan-temozolomide [BIT]). The allocation ratio was 1:1:1:1. In 2015, after release of results of the Innovative Therapies for Children with Cancer (ITCC) phase II trial of TTo,^11^ the trial was amended to evaluate a third chemotherapy regimen (TTo), and the design was changed to a 3 × 2 factorial trial (allocation ratio 1:1:1:1:1:1). Subsequently, when the accrual target for the IT arm was reached, the trial reverted to a 2 × 2 factorial design. Trial arms and details of chemotherapy doses are depicted in Figure 1 and Data Supplement (Table S1). The following minimization factors were used to balance all random assignments: (1) disease status (early relapse [defined as <18 months from the time of diagnosis] and late relapse [≥18 months] and refractory) and (2) measurable versus evaluable disease (as per RECIST v1.1). Interactions between treatment arms (ie, I and B) were not anticipated but were to be explored at trial completion using heterogeneity tests.

Trial treatment was given for six cycles. Patients with acceptable toxicities and response equal to or better than stable disease after six cycles were allowed to continue trial treatment for 12 cycles.

### Outcome Measures and Assessments

The primary outcome measure was initially overall response (complete or partial response) during the first six courses of trial therapy, evaluated locally at trial sites. Response was evaluated using RECIST 1.1 for patients with measurable disease.^23^ For those with only evaluable disease, response was evaluated using the new draft International Neuroblastoma Response Criteria including data on MIBG scores and bone marrow disease, which were subsequently published.^24^

After completing planned recruitment for the first 106 patients and meeting the trial-defined success criteria, the Independent Data Monitoring Committee (IDMC) recommended expansion of the B random assignment to 160 patients to include progression-free survival (PFS) as a coprimary outcome measure.

Secondary outcome measures were toxicity of the regimens (per Common Terminology Criteria Adverse Events v4.0), PFS, and overall survival (OS).

Response was evaluated at baseline and every two cycles using cross-sectional imaging with computed tomography/magnetic resonance imaging and I^123^ MIBG scans (or fluorodeoxyglucose-positron emission tomography scans if non-MIBG avid). Bilateral bone marrow aspirates and trephine biopsies were mandatory at baseline and repeated every two cycles in patients with bone marrow involvement at study entry or clinical suspicion of progression. All patients underwent mandatory imaging of the brain before study entry.

### Trial Design and Statistical Analysis

Efficacy data were analyzed on an intention-to-treat basis, and safety data were reported for all patients who received at least one dose of a trial drug. Trial sample size was calculated to detect a 15% increase in response rate with B. Assuming a baseline overall response rate (ORR) of 25% without B, using a two-stage Minimax Jung design with α and β errors set to .2, 42 patients per arm were required for the first stage and 53 patients per arm were required in total. Predefined success criteria for the trial were ≥4 more responses in the B arm compared with the non-B arm. Allowing for patients with response not assessed, the planned sample size was n = 120. Following a recommendation of the study IDMC, the B random assignment was then expanded to include PFS as coprimary end point: Assuming 40% PFS at 1 year in the control arm, with 160 patients and 80 events in total, there will be 80% power to detect a difference of 15% with a Cox proportional hazards model using a one-sided test with α = .15. All statistical analyses performed were predefined in the BEACON Statistical Analysis Plan.

For the primary end point of PFS for the expanded B random assignment, an adjusted hazard ratio (HR) with 95% CI was produced using a Cox proportional hazards model stratified for the other treatment allocation (I or topotecan or neither). The posterior probability distribution was then plotted for PFS, using a noninformative prior, on the basis of the HR calculated from the Cox model and the number of events observed.

For all random assignments, response was a primary endpoint. For response, risk ratios (RRs) with 95% CIs were calculated using logistic regression to compare responses (complete response/partial response) between arms for each random assignment. For the chemotherapy random assignments, Bayesian Beta-Binomial conjugate analyses were used to compare treatments by derived posterior distributions for the RR. Data in each arm are assumed to follow binomial distribution. A uniform prior β (1, 1) was used for the analysis. These plots are available to view in the Data Supplement (Figs S1-S3).

Toxicity, PFS, and OS were secondary end points. Toxicity was analyzed descriptively by presenting grade ≥3 adverse events (AEs) in each random assignment and those occurring in ≥1% of patients. Adjusted HRs with CI for the treatment effect for both PFS and OS outcomes were produced by implementing a Cox proportional hazards regression model for each treatment group (ie, for B and non-B, IT *v* T, and TTo *v* T), stratified for other treatment allocation. Kaplan-Meier survival curves were then produced for PFS and OS and survival estimates at 6, 12, and 24 months, and median survival time with 95% CIs was calculated. Heterogeneity tests in the form of forest plots were used for the PFS and OS outcomes to assess any potential interaction between arms (Data Supplement, Fig S1). Clinical trial protocol and statistical analysis plan are available as Data Supplement.

### Trial Oversight

This trial was performed in accordance with the principles of the Declaration of Helsinki and Good Clinical Practice guidelines. The trial obtained first competent authority and ethics committee approval in the United Kingdom (West Midlands—Coventry & Warwickshire Research Ethics Committee, reference 13/WM/0023) and then obtained competent authority and ethics committee approval in all the countries where it opened. The trial was designed by the Trial Management Group and the sponsor, the University of Birmingham, in collaboration with investigators from the ITCC and SIOPEN cooperative groups. Roche provided B supply and distribution. The authors attest to the accuracy and completeness of the data and the fidelity of the trial to the protocol. The trial was registered before the first patient recruited on April 24, 2013 (ISRTCN40708286; Eudract2012-000072-42; ClinicalTrials.gov identifier: NCT02308527).

## RESULTS

### Screening and Random Assignment

Between July 8, 2013, and February 7, 2019, a total of 160 eligible patients were randomly assigned from 43 sites in 10 European countries (sites are detailed in Appendix Table A1, online only): T (36), IT (30), TTo (14), BT (34), BIT (30), and BTTo (16). One hundred sixty patients were included in the B random assignment, 121 in the I random assignment, and 61 in the topotecan random assignment. Figure 1 depicts the trial CONSORT diagram.

### Patient Characteristics and Treatments

Age at enrollment ranged from 1 to 21 years (median, 5; range, 1-21). Baseline characteristics and prior therapies are depicted in Table 1 and the Data Supplement (Tables S2-S4): 93 patients (58%) had relapsed and 67 (42%) had refractory disease; 111 (69%) had measurable and 49 (31%) evaluable disease at study entry; 36 (23%) had tumors with *MYCN* amplification, and 64 (40%) had prior anti-GD2 immunotherapy.

**TABLE 1.** Patient Characteristics for the B Randomization

Characteristic	B (n = 80)	Non-B (n = 80)	Overall (N = 160)
Age, years, median (range)	5 (1-21)	5 (1-18)	5 (1-21)
Age, No. (%)			
<2	4 (5)	5 (6)	9 (5)
2 to <6	38 (47)	43 (54)	81 (51)
6 to <12	28 (35)	28 (35)	56 (35)
12 to <18	7 (9)	3 (4)	10 (6)
>18	3 (4)	1 (1)	4 (3)
Initial INSS stage, No. (%)			
1	1 (1)	1 (1)	2 (1)
2	3 (4)	0 (0)	3 (2)
3	4 (5)	6 (7.5)	10 (6)
4	68 (85)	69 (86)	137 (86)
4S	2 (2.5)	2 (2.5)	4 (2.5)
NA	0 (0)	1 (1)	1 (0.5)
Not known	2 (2.5)	1 (1)	3 (2)
*MYCN* amplification, No. (%)			
Present	20 (25)	16 (20)	36 (22)
Absent	58 (72)	62 (77)	120 (75)
Unknown	2 (3)	2 (3)	4 (3)
Induction chemotherapy, No. (%)			
COJEC	25 (31)	21 (26)	46 (29)
N7	5 (6)	4 (5)	9 (5)
TVD (after induction)	16 (20)	11 (14)	27 (17)
Other	14 (18)	12 (15)	26 (16)
Missing	20 (25)	32 (40)	52 (33)
Prior major surgery, No. (%)	58 (73)	53 (66)	111 (69)
High-dose chemotherapy with autologous stem-cell rescue, No. (%)	47 (59)	46 (58)	93 (58)
Prior radiotherapy, No. (%)	42 (53)	44 (55)	86 (54)
Prior anti-GD2 therapy, No. (%)	32 (40)	32 (40)	64 (40)
Performance status, No. (%)			
Lanksy			
90-100	58 (73)	63 (79)	121 (76)
70-80	10 (12)	6 (8)	16 (10)
50-60	5 (6)	2 (2)	7 (4)
Missing	7 (9)	9 (11)	16 (10)
Karnofsky			
90-100	6 (7)	3 (4)	9 (6)
70-80	2 (3)	2 (2)	4 (2)
Missing	72 (90)	75 (94)	147 (92)
Disease status^a^, No. (%)			
Refractory	34 (42)	33 (41)	67 (42)
Early relapse	31 (39)	32 (40)	63 (39)
Late relapse	15 (19)	15 (19)	30 (19)
Disease evaluation at study entry, No. (%)			
Measurable disease	56 (70)	55 (69)	111 (69)
Evaluable disease	24 (30)	25 (31)	49 (31)

Abbreviations: B, bevacizumab; COJEC, cisplatin, vincristine, carboplatin, etoposide, and cyclophosphamide; ECOG, Eastern Cooperative Oncology Group; INSS, International Neuroblastoma Staging System; I^123^-mIBG, metaiodobenzylguanidine; NA, not applicable; SIOPEN, European Neuroblastoma Research Network; TVD, topotecan, vincristine, doxorubicin.

^a^ Refractory disease was defined using the SIOPEN definition at the time of trial conduct: persistence of three or more spots on I^123^. MIBG scan (with or without persistence of bone marrow disease). Early relapse was defined as those occurring <18 months from the time of diagnosis and late relapse those occurring ≥18 months from the time of diagnosis.

The median follow-up for survivors was 1.5 years (IQR, 0.7-3.2 years; Data Supplement, Fig S2). Sixteen (10%) patients had response not evaluable and were considered nonresponders. Of them, six patients did not receive study treatment: one was ineligible post-random assignment, one experienced an AE, three withdrew from the study, and one patient progressed before treatment started. For the 10 patients who received study treatment, one patient had an unexpected change in diagnosis at cycle 2, six patients progressed before cycle 2 and one patient at cycle 2, and two patients died before cycle 2. For the present analysis, all 16 patients with response not assessed are considered nonresponders.

### B Random Assignment (T, IT, TTo *v* BT, BIT, BTTo)

Objective responses were seen in 21 of 80 patients (ORR, 26% [95% CI, 17 to 37]) in the B arms, and in 14 of 80 patients (ORR, 18% [95% CI, 10 to 28]) in the non-B arms (Table 2), the RR was 1.52 (95% CI, 0.83 to 2.77; *P* = .17). The predefined success criterion for ORR of *P* < .2 was met.

**TABLE 2.** Evaluation of Best Response to Trial Treatment According to Each Random Assignment

Response	B Random Assignment, No. (%)		I Random Assignment, No. (%)		Topotecan Random assignment, No. (%)	
	B (BT, BIT, BTTo; n = 80)	Non-B (T, IT, TTo; n = 80)	I (IT, BIT; n = 60)	No I (T, BT; n = 61)	Topotecan (TTo, BTTo; n = 30)	No Topotecan (T, BT; n = 31)
CR	6 (7)	1 (1)	4 (7)	0 (0)	2 (7)	1 (3)
PR	15 (19)	13 (16)	8 (13)	13 (21)	6 (20)	6 (19)
MR	2 (2)	1 (1)	3 (5)	0 (0)	0 (0)	0 (0)
SD	35 (44)	32 (40)	31 (51)	25 (41)	9 (30)	10 (33)
PD	15 (19)	23 (29)	10 (17)	15 (25)	9 (30)	11 (35)
Other/unknown	7 (9)	10 (13)	4 (7)	8 (13)	4 (13)	3 (10)
ORR, % (95% CI)	26 (17 to 37)	18 (10 to 28)	20 (11 to 32)	21 (12 to 34)	27 (12 to 46)	23 (10 to 41)

Abbreviations: B, bevacizumab; BIT, bevacizumab-irinotecan-temozolomide; BT, bevacizumab-temozolomide; BTTo, bevacizumab-topotecan-temozolomide; CR, complete response; I, irinotecan; IT, irinotecan-temozolomide; MR, minor response; ORR, overall response rate (complete + partial response); PR, partial response; PD, progressive disease; SD, stable disease; T, temozolomide; TTo, topotecan-temozolomide.

**FIG 2.**
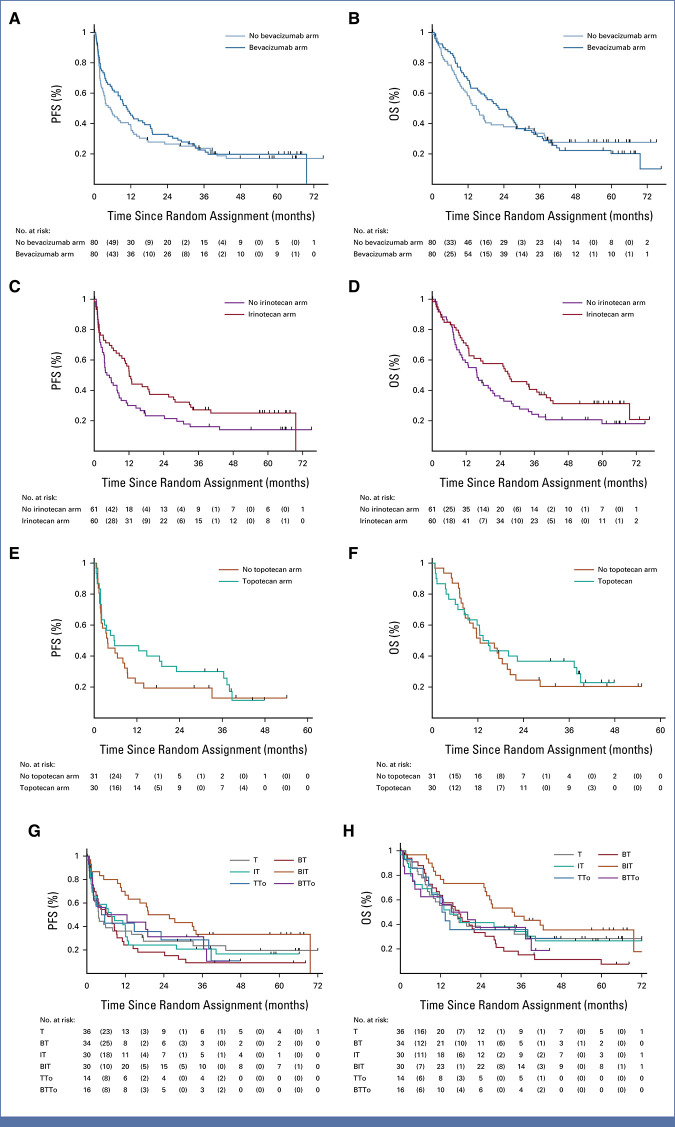
Survival analysis in the BEACON Neuroblastoma trial. Left panels show progression-free survival and right panels show overall survival. (A and B) Bevacizumab random assignment. (C and D) Irinotecan random assignment (T [no irinotecan] *v* IT [irinotecan]). (E and F) Topotecan random assignment (T [no topotecan] *v* TTo [topotecan]). (G and H) Survival for all trial arms. BT, bevacizumab-temozolomide; BIT, bevacizumab-irinotecan-temozolomide; BTTo, bevacizumab-topotecan-temozolomide; IT, irinotecan-temozolomide; OS, overall survival; PFS, progression-free survival; T, temozolomide; TTo, topotecan-temozolomide.

The HR for PFS was 0.89 (95% CI, 0.63 to 1.27). The 1-year PFS in the B arms was 0.46 (95% CI, 0.34 to 0.56) compared with 0.38 (95% CI, 0.27 to 0.49) in the non-B arms. PFS and OS for all random assignments and arms are shown in Figure 2.

The HR for OS was 1.01 (95% CI, 0.70 to 1.45). The 1-year OS was 0.69 (95% CI, 0.57 to 0.77) for B arms compared with 0.58 (95% CI, 0.47 to 0.68) for non-B arms.

The posterior plot for PFS for the B random assignment derived from the HR, number of events, and a noninformative prior β (0.1, 0.1) is shown in the Data Supplement (Fig S3). The probability, given the data, of the true HR being <1, that is, in favor of B treatment, is 73%.

### I Random Assignment (T, BT *v* IT, BIT)

Objective responses were seen in 12 of 60 patients (ORR, 20% [95% CI, 11 to 32]) in the IT arms and 13 of 61 patients (ORR, 21% [95% CI, 12 to 34]) in the T arms (Table 2). The RR was 0.94 (95% CI, 0.47 to 1.89).

The HR for PFS was 0.59 (95% CI, 0.39 to 0.90) for patients in IT arms compared with those receiving T. The 1-year PFS was 0.53 (95% CI, 0.39 to 0.64) for IT arms compared with 0.30 (95% CI, 0.19 to 0.42) for patients receiving T only.

The HR for OS was 0.65 (95% CI, 0.43 to 0.99) for patients in IT arms compared with those receiving T. The 1-year OS was 0.70 (95% CI, 0.56 to 0.80) for IT arms compared with 0.58 (95% CI, 0.45 to 0.70) for patients receiving T only (Table 2).

A posterior distribution for the RR was derived by performing simulations on the number of responses and noninformative β (0.1, 0.1) prior for the I random assignment as shown in the Data Supplement (Fig S3). An RR of 0.939 (95% credible interval [CrI], 0.46 to 1.90) was obtained. A normal distribution curve is shown for comparison. The posterior probability of true RR >1.0, 1.2, 1.4, and 1.6 is presented.

### Topotecan Random Assignment (T, BT *v* TTo, BTTo)

Objective responses were seen in 8 of 30 patients (ORR, 27% [95% CI, 12 to 46]) in TTo arms and 7 of 31 patients (ORR, 23% [95% CI, 10 to 41]) in T arms. The RR was 1.22 (95% CI, 0.51 to 2.94).

The HR for PFS was 0.59 (95% CI, 0.33 to 1.08) for patients in TTo arms compared with those receiving T. The 1-year PFS was 0.47 (95% CI, 0.28 to 0.63) for patients receiving TTo compared with 0.23 (95% CI, 0.10 to 0.38) for patients in T arms.

The HR for OS was 0.80 (95% CI, 0.44 to 1.45) for patients in TTo arms compared with those receiving T. The 1-year OS was 0.60 (95% CI, 0.40 to 0.75) in TTo arms compared with 0.52 (95% CI, 0.33 to 0.67) in T arms.

A posterior distribution for the RR was derived by performing simulations on the number of responses and noninformative β (0.1,0.1) prior for the topotecan random assignment as shown in the Data Supplement (Fig S3). An RR of 1.181 (95% CrI, 0.48 to 3.02) was obtained. A normal distribution curve is shown for comparison. The posterior probability of true RR >1.0, 1.2, 1.4, and 1.6 is presented.

### Potential Interactions

The trial design assumed no interaction between B and the different chemotherapy regimens. At trial completion, interactions were explored by heterogeneity tests. The unadjusted heterogeneity test for PFS showed some evidence of a potential interaction between irinotecan and B (*P* = .11; Data Supplement, Fig S1A1), but not for OS nor for topotecan (Data Supplement, Figs S1A2 and S1B-S1K). PFS and OS for all treatment arms are shown in Figures 2G and 2H. One-year and 2-year PFS estimates for patients in the BIT arm were 0.67 (95% CI, 0.47 to 0.80) and 0.50 (95% CI, 0.31 to 0.66), respectively. One-year and 2-year OS estimates for patients in the BIT arm were 0.77 (95% CI, 0.57 to 0.88) and 0.73 (95% CI, 0.54 to 0.86), respectively. No formal statistical comparisons were planned between individual trial arms.

### Safety: B Random Assignment

Grade 3 or worse AEs are shown in Table 3 and the Data Supplement (Table S5). Patients receiving B experienced more AEs than those not receiving it. More patients in the B-receiving arms experienced neutropenia, anemia, and thrombocytopenia. Ten patients receiving B experienced proteinuria. Grade ≥3 proteinuria occurred in four patients (5%) receiving B and no patients receiving chemotherapy alone. No patients had episodes of grade ≥3 bleeding, wound healing complications, fistulae, posterior reversible encephalopathy syndrome, congestive heart failure, thromboembolic events, or GI perforation.

**TABLE 3.** Grade ≥3 AEs in the B Random Assignment Occurring in ≥1% of Patients

CTCAE Category	B Random Assignment (grade 3 and above AEs)		
	B (BT, BIT, BTTo; n = 80), No. (%)	Non-B (T, IT, TTo; n = 80), No. (%)	Overall (N = 160), No. (%)
Investigations	218 (59)	190 (50)	408 (54)
Neutrophil count decreased	77 (30.5)	82 (23.5)	159 (51.5)
Platelet count decreased	86 (23)	68 (21)	154 (21)
WBC decreased	23 (4)	14 (5)	37 (9)
Lymphocyte count decreased	18 (8)	13 (3)	31 (10)
GGT increased	2 (1)	2 (2.5)	4 (2)
ALT increased	5 (2.5)	9 (2.5)	14 (2.5)
AST increased	1 (1)	4 (2.5)	5 (2)
Blood and lymphatic system disorders	32 (19)	23 (8)	55 (13)
Anemia	25 (14)	17 (3)	44 (16)
Febrile neutropenia	4 (5)	2 (3)	6 (4)
Metabolism and nutrition disorders	24 (14)	12 (6)	36 (10)
Anorexia	5 (4)	8 (4)	13 (4)
Dehydration	6 (6)	0 (0)	6 (3)
GI disorders	21 (15)	13 (10)	34 (13)
Diarrhea	12 (5)	2 (1)	14 (3)
Vomiting	3 (4)	5 (2.5)	8 (3)
Nausea	2 (2.5)	1 (1)	3 (1)
Abdominal pain	0 (0)	2 (2.5)	2 (1)
Infections and infestations	20 (13)	9 (9)	29 (11)
Catheter-related infection	7 (6)	1 (1)	8 (4)
Abdominal infection	3 (2.5)	0 (0)	3 (1)
Lung infection	1 (1)	2 (2.5)	3 (2)
Varicella	2 (2.5)	0 (0)	2 (1)
Upper respiratory infection	0 (0)	2 (2.5)	2 (1)
General disorders and administration site conditions	7 (5)	14 (5)	21 (5)
Fever	2 (2.5)	5 (4)	7 (3)
Pain	3 (2.5)	4 (4)	7 (3)
Renal and urinary disorders	10 (4)	0 (0)	10 (2)
Proteinuria	8 (4)	0 (0)	8 (2)
Total No. of AEs	350	273	623

NOTE. Only two patients experienced grade 5 toxicities during the trial: worsening general condition and pulmonary edema.

Abbreviations: AE, adverse event; B, bevacizumab; BIT, bevacizumab-irinotecan-temozolomide; BT, bevacizumab-temozolomide; BTTo, bevacizumab-topotecan-temozolomide; CTCAE, Common Terminology Criteria Adverse Events; GGT, gamma-glutamyl transferase; IT, irinotecan-temozolomide; T, temozolomide; TTo, topotecan-temozolomide.

### Safety: Chemotherapy Random Assignments

Patients receiving irinotecan or topotecan experienced more AEs than those receiving T alone (Data Supplement, Table S6). Patients receiving irinotecan had an increased incidence of ≥grade 3 GI AEs, mainly diarrhea in eight patients (13%). Patients receiving topotecan had an increased incidence of grade ≥3 neutropenia, thrombocytopenia, and anemia.

## DISCUSSION

To our knowledge, we report the largest randomized trial in relapsed/refractory neuroblastoma conducted to date. Using a multiarm multistage factorial design, three chemotherapy regimens and a novel agent, B, added to conventional chemotherapy, were evaluated within one international cooperative group academic clinical trial.

At the time of designing the trial, there was considerable uncertainty as to which of the three chemotherapy regimens was superior for the treatment of relapsed/refractory neuroblastoma; all had shown promising activity in single-arm series with similar response rates and were commonly used internationally depending on physician preference.^9-11^ Identifying the most appropriate backbone chemotherapy regimen is critical to evaluate novel combinations for future therapy, and only a randomized controlled comparison can provide the necessary data.

The addition of irinotecan or topotecan to T did not achieve higher response rates but appeared to result in improved PFS. One-year PFS seemed higher in irinotecan- or topotecan-containing arms (23% and 24% higher, respectively) compared with T-only arms. Toxicity profiles confirmed that the addition of irinotecan is associated with more diarrhea and GI symptoms, whereas the addition of topotecan is associated with increased myelotoxicity. Whether one or the other toxicity profile is preferable remains a subject of debate and may vary from patient to patient. The trial did not include quality-of-life questionnaires or patient-reported outcome measures, which would have been extremely useful to base future choices incorporating the patients' and parents' voices. In the B random assignment of the BEACON trial, success criteria were met for response. PFS seemed to improve with the addition of B within the limitations of evidence generated within a randomized phase II trial. The addition of B also increased toxicity, mainly related to increased myelotoxicity. Consistent with other large trials in the pediatric population, B-specific toxicities seen in adults were rare, although grade 3-4 proteinuria occurred in 5% of patients.^25,26^

An unexpected potential interaction between irinotecan and B was seen, with possibly greater benefit of B in patients also receiving irinotecan. This interaction is biologically plausible: Although B is primarily an antiangiogenic agent, it has a number of effects on the tumor immune microenvironment, including promotion of tumor-infiltrating lymphocytes, with suppression in the number and function of inhibitory populations, including myeloid-derived suppressor cells, regulatory T cells, and M2 macrophages.^27-29^ Irinotecan has also been reported to have immune and antiangiogenic effects, including depletion of regulatory T cells, upregulation of major histocompatibility complex class I, and PD-L1 expression.^30-32^ The benefits of randomized comparisons are highlighted as the results of the BEACON trial differ from a single-institution single-arm trial which showed no benefit of adding B to IT in patients with highly pretreated neuroblastoma.^17^

The unexpected potential interaction identified after completing the trial highlights one of the challenges of using factorial designs. The strength of this design was that it allowed four different questions to be addressed within the same trial population and generated randomized phase II data.

The current results need to be analyzed in the context of recently reported trials in the relapsed/refractory neuroblastoma setting. The combination of anti-GD2 therapy with chemotherapy has shown very promising results in single-arm studies and smaller randomized trials.^33-35^ In the Children's Oncology Group ANBL1221 trial, 53 children were treated with IT-dinutuximab-granulocyte-macrophage colony-stimulating factor, with an ORR of 41.5% and 1-year PFS of 67.9%. After completing the B random assignment, the BEACON trial was amended to evaluate the addition of anti-GD2 therapy with dinutuximab beta to chemotherapy.

Despite promising results, a significant proportion of patients still relapsed. Better understanding of the biology of refractory/relapsed and identifying biomarkers of response or resistance to therapy remain crucial. An ambitious ongoing translational research program (BEACON BIO) is currently analyzing more than 700 plasma, blood, and tumor samples collected during trial treatment.^36^

In conclusion, within the limits of a phase II trial, the BEACON trial provided some evidence that addition of B to T-based chemotherapy improved ORR. It also appeared to show some improvement PFS when adding irinotecan or topotecan to T. A potential interaction between irinotecan and B might explain better results for the BIT regimen. A future study in relapsed/refractory neuroblastoma will compare IT-B with a chemoimmunotherapy regimen.

## APPENDIX

**TABLE A1.** List of Trial Sites and Innovative Therapies for Children with Cancer (ITCC) and European Association for Neuroblastoma Research (SIOPEN) Investigators

Site Name	Principal Investigator	City	Country	No. of Patients
Institut Gustave Roussy	Dominique Valteau-Couanet	Villejuif	France	13
Hospital del Nino Jesus	Francisco J. Bautista	Madrid	Spain	9
Royal Hospital for Sick Children (Yorkhill)	Dermot Murphy	Glasgow	The United Kingdom	9
Royal Manchester Children's Hospital	Guy Makin	Manchester	The United Kingdom	9
Hôpital Mère-Enfant, Nantes	Estelle Thebaut	Nantes	France	8
Princess Maxima Centrum	Natasha van Eijkelenburg	Utrecht	The Netherlands	8
Birmingham Children's Hospital	Dave Hobin	Birmingham	The United Kingdom	8
The Royal Marsden Hospital	Sucheta Vaidya	London	The United Kingdom	8
Leeds General Infirmary	Martin Elliott	Leeds	The United Kingdom	7
Institut Curie	Gudrun Schleiermacher	Curie	France	6
Our Lady Children's Hospital	Cormac Owens	Dublin	Ireland	6
Great Ormond Street Hospital	Giuseppe Barone	London	The United Kingdom	6
Royal Victoria Infirmary	Deborah Tweddle	Newcastle	The United Kingdom	6
Instituto de Investigación Sanitaria La Fe	Victoria Castel	Valencia	Spain	5
Righospitalet	Karsten Nysom	Copenhagen	Denmark	4
Hôpital des Enfants	Julie Tandonnet	Bordeaux	France	4
Centre Leon Berard	Nadege Corradini	Lyon	France	4
Hôpital d'Enfants	Pascal Chastagner	Nancy	France	4
Hôpital des Enfants	Marion Gambart	Toulouse	France	4
Hôpital de Hautepierre	Sarah Jannier	Strasbourg	France	4
Bambino Gesù Children's Hospital	Aurora Castellano	Rome	Italy	4
Hospital Vall d'Hebron	Soledad Gallego	Barcelona	Spain	4
Hôpital de la Timone	Carole Coze	Marseille	France	3
Hôpital Armand Trousseau	Anne Auvrignon	Trousseau	France	3
Center Oscar Lambret	Anne Sophie Defachelles	Lille	France	2
Hospital de Cruces	Ricardo Lopez Almaraz	Bilbao	Spain	2
University Children's Hospital	Nicolas Gerber	Zurich	Switzerland	2
St Anna Kinderspital	Ruth Ladenstein	Vienna	Austria	1
Universitair Hospital Gent	Bram De Wilde	Gent	Belgium	1
Cliniques Universitaires Saint Luc (UCL)	Benedicte Brichard	UCL	Belgium	1
Istituto Nazionale Tumori	Roberto Luksch	Milan	Italy	1
Erasmus Medical Centre/Sophia Children's Hospital	C. Michel Zwaan	Rotterdam	The Netherlands	1
Centre Hospitalier Universitaire Vaudois	Maja Beck-Popovic	Lausanne	Switzerland	1
Royal Aberdeen Children's Hospital	Courtney Willis	Aberdeen	The United Kingdom	1
University Hospital Southampton	Juliet Gray	Southampton	The United Kingdom	1
University Children's Hospital Freiburg	Simone Hettmer	Freiburg	Germany	0
HELIOS Klinikum Berlin-Buch	Lothar Schweigerer	Berlin	Germany	0
Universitatsklinikum Aachen	Udo Kontny	Aachen	Germany	0
University Hospital Leipzig	Holger Christiansen	Leipzig	Germany	0
Giannina Gaslini Children's Hospital	Alberto Garaventa	Genoa	Italy	0
Bristol Royal Hospital for Children	Anthony Ng	Bristol	The United Kingdom	0
Alder Hey Children's Hospital	Lisa Howell	Liverpool	The United Kingdom	0
Sheffield Children's Hospital	Daniel Yeomanson	Sheffield	The United Kingdom	0
		Total		160
